# Comparative Experimental Study of Dermal Stability: Acellular Dermal Matrix versus Crayopreserved Dermis

**DOI:** 10.29252/wjps.10.2.82

**Published:** 2021-05

**Authors:** Mahmood Omranifard, Mehdi Rasti Ardakani, Hossein Abdali, Pejman Mortazavi, Saeed Hoseini, Mohammad Ali Hoghoughi

**Affiliations:** 1 Department of Plastic Surgery, School of Medicine, Isfahan University of Medical Sciences, Isfahan, Iran; 2 Department of Pathology, School of Specialize Veterinary Sciences, Science and Research Branch, Islamic Azad University, Tehran, Iran; 3 Burn and Wound Healing Research Center, Shiraz University of Medical Science, Shiraz, Iran

**Keywords:** Inflammation, Acellular dermal matrix, Crayopreserved dermis, Collagen, Fibroblasts, Pathological neovascularization

## Abstract

**BACKGROUND:**

Given the potential usefulness of Acellular Dermal Matrices (ADM) for wound healing, we aimed to evaluate the stability, histological characteristics, and effectiveness of ADM compared with cryopreserved dermis (CPD) in rat models.

**METHODS:**

This experimental study was conducted in the Department of Surgery, Isfahan University of Medical Sciences, Isfahan, Iran, from January to March 2015. The prepared ADM and CPD were transplanted to the full-thickness skin defects on the back of Sprague-Dawley rats. Forty-five days after grafting, the tissues were harvested for histological examination. These two types of the dermis' quality and stability were compared with consideration of the following factors; inflammation, fibroblasts migration, vascularization, collagen formation, capsule formation, and microabscess formation.

**RESULTS:**

From 19 selected rates, nine received CPD, and ten were treated with ADM. After transplantation, the mean (SD) weight of ADM and CPD grafts were 1.74 (0.07) and 1.45 (0.77), respectively (*P*<0.001). The frequency of inflammation was significantly higher in CPD grafts (*P*<0.01). Higher grades of collagen organization, fibroblast spreading, and vascularization were more frequent in ADM grafts (*P*<0.01). The frequency of capsule and microabscesses formation was not significantly different between studied groups.

**CONCLUSION:**

ADM have a superior effect than CPD in the wound healing process. Both samples had a similar effect in capsule and microabscesses formation and higher costs of ADM preparation. According to the physicians' decision and evaluation of the process's cost-effectiveness, CPD could be appropriately used as an alternative to ADM.

## INTRODUCTION

Dermal substitutes are considered strategies developed for better management of cutaneous wounds and disease. They promote skin regeneration and the quality of wound healing by covering the lesion and facilitating cell colonization^1^. Skin substitutes could be used as an appropriate alternative to skin grafts^2^.

Dermal substitutes serve as scaffolding that supports the infiltration of fibroblasts and endothelial cells, neovascularization, and epithelialization and consequently enhance neodermis formation during the wound healing process^3^^,^^4^.

Different manufacturing methods with various physical characteristics such as decellularization, sterilization, freeze-drying, and crosslinking protocols have been used for processing scaffolds^5^. From mentioned techniques, the decellularized dermis was the best option with more clinical results^6^.

 Some studies showed the utility of Acellular Dermal Matrices (ADM) and their clinical application compared to other cell-containing bilayered skin substitute constructs^7^^,^^8^. There are also reports regarding the cost-effectiveness and reduced hospitalization of ADM, especially in surgical procedures^9^^,^^10^.

ADM has been used to repair genital and in post-burn injuries, abdominal, breast, head and neck reconstruction, and cancer, ulcers, and post-trauma surgery^11^^-^^15^.

Given the potential usefulness of ADM for wound healing, this study aimed to evaluate the stability, histological characteristics, and effectiveness of ADM compared with cryopreserved dermis (CPD) in the vascularization, fibroblast migration, inflammation and collagen organization in rat models. However, our findings would provide us important information for better management of cutaneous wounds and disease as well as developing more effective therapeutic strategies for skin regeneration.

## MATERIALS AND METHODS

This experimental study was conducted in the Department of Surgery, Isfahan University of Medical Sciences, Isfahan, Iran, from January to March 2015.

The study's protocol was approved by the Regional Ethics Committee of Isfahan University of Medical Sciences (research project number; 394209). Before resectioning the skin, written informed consent was obtained from the women after explaining our study's aims.

During this study, a normal human skin was used for preparing ADM and CPD. The prepared ADM and CPD were transplanted to the full-thickness skin defects on the back of Sprague-Dawley rats. About 45 d after grafting, the tissues were harvested for histological examination. These two types of dermis' quality and stability were compared with consideration of the following factors; inflammation, fibroblasts spreading, vascularization, collagen formation, capsule formation, and microabscess formation.

*Source of skin tissue*

Regarding the dermis source, normal human skin was provided from the skin of multiparous women with large pannus in the lower abdomen, aged 48 yr old, who underwent a high lateral tension abdominoplasty.

A full-thickness skin was excised under sterile conditions of the operation room. The skin was scrubbed, and the epidermis and subdermal fat tissue were entirely removed for providing a dermal matrix (DM) with a thickness of 0.20 mm. The DM transversely bisected, and one part was assigned for cryopreserve dermis preparation and the other for ADM preparation. The samples were wrapped in phosphate-buffered saline solution (0.01MPBS, PH=7.4), freezed, and stored at -70 °C.

The cryopreserved dermis sample was stored with any additional procedure, and the other stored until acellular process as described below.

*ADM preparation*

The ADM was prepared by a modified method, described previously^16^. The frozen reticular DM samples were thawed and soaked in double-distilled water for 30 min at room temperature (22-24 °C).

The samples were treated with 0.25% (w/v) trypsin (Sigma, Cas: Y4799)/EDTA–Na_2_ at 37 °C for 2 h with continuous shaking (80 rpm), and were thoroughly rinsed in PBS for three times (15 min each) at 22-24 °C. Then, they were incubated in lysis buffer (Triton X-100, NaCl, Tris HCl, DDT, Glycerol) for 24 h at 22–24 °C with continuous shaking to remove cellular components from the dermal matrix. In the last step, the samples were thoroughly rinsed in PBS and Sodium Azide at room temperature three times (15 min each). The prepared ADM samples were then placed in sterile plastic bags and stored at −20 °C until the day of the animal experiment. All solutions were filter sterilized, and all procedures were performed aseptically.

**Fig. 1:**
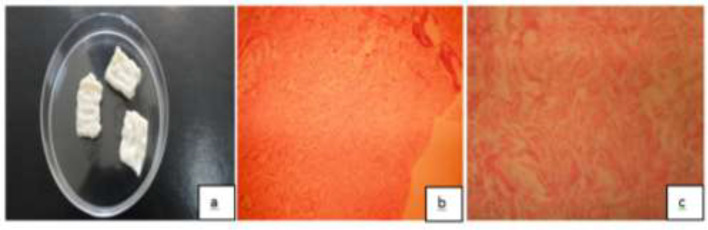
Acellular dermal matrices(ADM) and cryopreseved dermis(CPD) prepation;a: ADM samples, b: CPD freezed and stored at -70°C[(0.01MPBS,PH=7.4),( H&E*160)],c: ADM prepared by a modified methods(H&E*160).

All prepared ADM samples were evaluated by an expert pathologist for verifying the procedure of ADM preparation (Figure1 a,b,c).

***Animal Experiments***

**Fig. 2:**
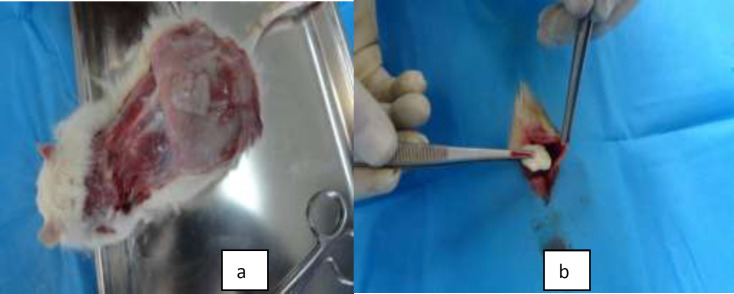
Animal experiments; a: transplantation of ADM and CPD samples to the subcutaneous pockets of the Sprague-Dawley rats neck, b: excision of the transplanted areas for macroscopic and histological evaluations (45 d after the grafting)

Twenty-two Sprague-Dawley rats were utilized. These rats aged 12-16 wk and weighing between 360 and 410 gr. They obtained from the Animal Center of Isfahan University of Medical Sciences. They were randomly divided into two groups. One group received CPD, and the other was treated with ADM. Under sterile conditions, they anesthetized by intraperitoneal administration of pentobarbital (w/v) 1% with a dose of 45 mg/kg. After shaving the hairs, a dermal flap (1 x 3 cm) was elevated on the neck of the studied rats and weighing a certain amount of ADM and CPD samples were transplanted to the sites(subcutaneous pockets of the rat's neck) (Figure 2 a).

Forty-five days after the grafting, the rats in each group were euthanized, the transplant area was excised and halved for macroscopic and histological evaluations (Figure 2 b). The quality and stability of the grafts and surrounding tissue were evaluated by determining the numerical density per area (NA) of fibroblasts, inflammatory cells, blood vessels, and the structure of collagen fibers, capsule formation and microabscess formation in a 45 μm2 frame, in each group.

The presence of fibroblasts, inflammatory cells, blood vessels, and the structure of collagen fiber bundles was classified according to the degree of presence of the cells in the histopathologic samples using the following scale: 0, non-presence; 1, less than 25%; 2.25%-50%; 3.50%-75% and 4, more than 75%.

***Statistical analysis***

Data were processed by SPSS statistical software program version 21 (SPSS Inc., Chicago, IL, U.S.A.). Student's t-test and Chisquare test were used to compare quantitative and qualitative variables, respectively. A P-value less than 0.05 was considered statistically significant.

## RESULTS

During this study, 3 of the 22 selected rats had not appropriated clinical and technical conditions for enrollment in the study, and 19 rats were finally enrolled. From the selected rates, 9 received CPD, and 10 treated with ADM. The mean weight of the grafts before transplantation was 2 mg in each group. After transplantation, the mean (SD) weight of ADM and CPD grafts were 1.74 (0.07) and 1.45 (0.77), respectively (*P*<0.001).

**Fig. 3:**
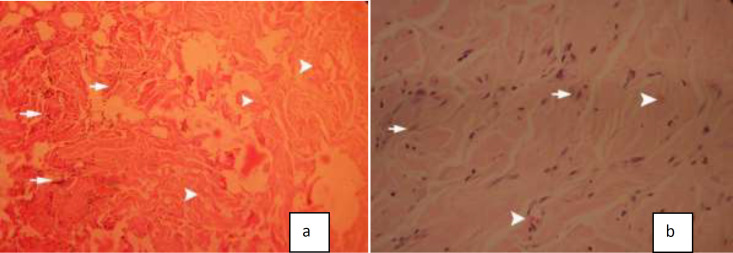
Histopathologic characteristics of cryopreserved dermis(CPD);a:higher expression of inflammatory cells and collagen in CPD grafts(H&E*64), b: less fibroblast spreading and less vascularization in CPD grafts(H&E*640).

**Fig. 4:**
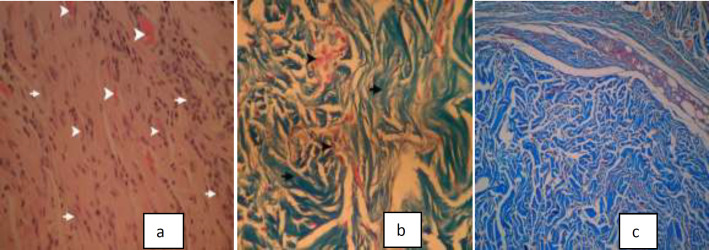
Histopathologic characteristics of acellular dermal matrices (ADM);higher grade of collagen organization, vascularization and fibroblast spreading in ADM[a: H&E*640,b: Trichrome*640],c:significant cellular repopularization and collagen organization in ADM ( Trichrome*64).

Histopathologic findings of the grafts and their surrounding tissue in two ADM and CPD groups are presented in Table 1. The frequency of inflammation and collagen degradation was significantly higher in CPD grafts (Figure 3a) with less fibroblast migration and vascularization (Figure 3b) (*P*<0.01). Higher grades of collagen organization, fibroblast spreading, and vascularization were more frequent in ADM grafts (Figure 4a and 4b) (*P*<0.01). We could see significant cellular repopularization and collagen organization in the ADM group (Figure 4c). The frequency of capsule and microabscesses formation was not significantly different between studied groups.

**Table 1:** Histopathologic characteristics of the grafts and their surrounding tissue in preparing acellular dermal matrix (ADM) and cryopreserved dermis (CPD) groups

*Variables*	*ADM* *n=10*	*CPD* *n=9*	*P-value*
**-Inflammatory cells** **Grade0** **Grade1** **Grade 2** **Grade 3** **Grade4**	3(30%) 5(50%) 2(20%) 0(0%) 0(0%)	0(0%) 1(11.1%) 1(11.1%) 3(33.3%) 4(44.4%)	0.01
**-Fibroblasts** **Grade0** **Grade1** **Grade 2** **Grade 3** **Grade4**	0(0%) 0(0%) 0(0%) 4(40%) 6(60%)	0(0%) 3(33.3%) 4(44.4%) 2(22.2%) 0(0%)	<0.001
**-Collagen organization** **Grade0** **Grade1** **Grade 2** **Grade 3** **Grade4**	0(0%) 0(0%) 0(0%) 2(20%) 8(80%)	0(0%) 5(55.6%) 3(33.3%) 1 (11.1%) 0(0%)	<0.001
**Number of vessels[mean(SD)]**	13.8(2.48)	4.0(1.22)	<0.001
**Capsule formation**	9(100%)	8(80%)	0.47
**Microabscesses formation**	0(0%)	0(0%)	1.00

## DISCUSSION

In this experimental study, we comparatively evaluated ADM's stability and CPD in a rat model skin wound. Higher grades of inflammation and collagen degradation were more prevalent in CPD than ADM samples, and higher grades of collagen organization, fibroblast spreading, and vascularization were more prevalent in ADM than CPD samples, 45 d after the procedure. There was not any significant difference between groups regarding capsule and microabscesses formation.

Several studies demonstrated the advantages of ADM use as dermal substitutes in different procedures, and some studies compare the characteristics of different products of ADM^17^^,^^18^. However, there were few similar studies, and most of them have indicated better satisfaction and outcome for ADM than frozen human cadaver allograft^19^^,^^20^. In this study, ADM had a lower grade of collagen degradation and inflammation comparing with CPD.

Collagen organization and neovascularization in different types of ADM products have been proved by many studies^21^^,^^22^. In this study, we observed a higher grade of vascularization, fibroblast spreading, and collagen organization in ADM than CPD samples.

The results of the current study indicated no cases of microabscess formation in the two studied groups. There was not any significant difference between the two groups. There is a report regarding the protective effects of ADM for microabscesses formation^23^.

ADM could inhibit capsule formation mainly by reducing myofibroblast activity. Further, there are reports regarding the protective effect of ADM in the capsular formation and capsular fibrosis, as well as decreasing fibroblast cellularity. Most of the mentioned reports were related to breast reconstruction^24^^, ^^25^.

In this study higher grade of fibroblast spreading was observed in ADM and lower grade in CPD group. In our study, the capsule formation rate was lower in ADM than CPD, though the difference was not statistically significant. After transplantation, the mean weight of ADM was significantly higher than CPD grafts. In order to explain our findings, it is necessary to review the different phases of bio integration in ADM, which is similar to normal wound healing.

The phases in order were inflammatory phase, fibroblast migration, neovascularization, remodeling and maturation. Migration of leukocytes and macrophages across the matrix is occurred during the inflammatory phase and fibroblast migration in remodeling phase, respectively. Migration of the cells during mentioned phases from wound borders to collagen networks results in the formation of a three-dimensional scaffold. A well-structured organization on hematic vessels is processed during neoangiogenesis. Finally, in the remodeling and maturation phase, matrix degeneration and biosynthesis of collagen are completed by fibroblasts. The results of these overlapping phases are restoring tissue integrity and homeostasis^26^^,^^27^.

The histopathologic evaluation in our study was performed 45 d after transplantation. Considering our findings, which indicated higher grades of inflammation and collagen degradation in CPD and higher grades of vascularization, fibroblast spreading and collagen organization in ADM samples, we could suggest that the process of wound healing in ADM and consequently wound stability is faster than CPD, because, at the time of evaluation, CPD samples are in the first phases of wound healing whereas ADM samples were in the last phases of wound healing. Thus, when the timing of wound healing is important such as diabetic ulcers or some burn wounds, using of ADM would provide us more appropriate outcomes. 

The limitations of the current study were the small sample size and short duration of the study. Moreover, in order to obtain more conclusive results regarding the progression of different phases of wound healing in CPD and ADM samples, it is recommended to have a frequent evaluation, for example, with weekly periods for clarifying different phases of wound healing.

## CONCLUSION

ADM have a superior effect than CPD in the wound healing process. On the other hand, considering that both samples had a similar effect in capsule and microabscesses formation and higher costs of ADM preparation, it seems that according to the physicians' decision and evaluating the cost-effectiveness of the process, CPD could be used properly as an alternative to ADM. 
